# In This Issue

**DOI:** 10.1002/cfg.481

**Published:** 2005-06


**Comparative and Functional Genomics**
*Comp Funct Genom* 2005; **6**: 193.
Published online in Wiley InterScience (www.interscience.wiley.com). **DOI:** 10.1002/cfg.481

### In this issue

### High resolution map of the bovine hornless locus

The hornless phenotype in cattle is of interest due to its economic importance. **Cord Drögemüller** *et al.* have produced a 4 Mb high resolution BAC contig of the bovine chromosome 1q12 region which contains the putative locus and used it to refine the comparative map with human chromosome 21q22.

### Cadherin repertoire of the mosquito, and comparison to the fruitfly

The cadherin superfamily is involved in cell-cell communication and adhesion and is widely represented across the evolutionary tree. **Catarina Moita and colleagues** have used a bioinformatic approach to identify the cadherin repertoire of *Anopheles gambiae* (the mosquito) and compared it to that of the fruitfly *Drosophila melanogaster*.

### Phylogenomic analysis of the alphavirus genus

Using full or partial genome sequence data from alphavirus genomes **Aimée Luers and coworkers** have made a phylogenomic study of the alphavirus genus. Their approach, which uses much more data for each virus than prior studies, has yielded some novel insights into the evolution of this genus.

### Special section of reviews and papers from the Plant and Animal Genomes XIII (PAGXIII) conference

**Ulrik John and colleagues** have termed their approach for identifying novel genes and alleles from indigenous and exotic species Xenogenomics. This genomic bioprospecting involves EST discovery, cDNA microarray-based expression profiling and functional genomics and is being applied to indigenous Australian flora and native Antarctic plants.

**Robert Moritz** *et al.* describe their novel prefractionation strategy, which uses two-dimensional free-flow electrophoresis. This technique can fractionate large quantity protein samples and is applicable to both high-$M_r$ proteins and small peptides. They have applied it to the proteomic analysis of colorectal cancer, to discover biomarkers for this disease.

Fatal bovine respiratory infections usually occur when a primary viral infection compromises host defences and enhances the severity of a secondary bacterial infection. **Paul Hodgson and colleagues** discuss their observations on the effect of stress on this viral-bacterial synergy, which appears to be enacted via regulation of inflammation.

**Lawrence Schook and coworkers** review the Swine Genome Sequencing Consortium (SGSC) workshop: A Strategic Roadmap for Sequencing the Pig Genome. Presentations reported the completion of a human-pig comparative map, and BAC fingerprint contigs for each of the autosomes and X chromosomes, which have been anchored using BLAST analysis and RH-mapping. Significant progress has been made towards the creation of a minimal tiling path for sequencing.

### Conference Calendar

A listing of genomics related conferences planned for October to December 2005.

Copyright © 2005 John Wiley & Sons, Ltd.

